# Can the CalproQuest predict a positive Calprotectin test? A prospective diagnostic study

**DOI:** 10.1371/journal.pone.0224961

**Published:** 2019-11-21

**Authors:** Corinne Chmiel, Oliver Senn, Susann Hasler, Thomas Rosemann, Gerhard Rogler, Nadine Zahnd, Ryan Tandjung, Nathalie Scherz, Michael Christian Sulz, Stephan Vavricka

**Affiliations:** 1 Institute of Primary Care, University and University Hospital of Zurich, Switzerland; 2 Department of Gastroenterology and Hepatology, University Hospital Zurich, Switzerland; 3 IBDnet, Swiss Research and Communication Network on Inflammatory Bowel Disease, Zurich, Switzerland; 4 Division of Gastroenterology and Hepatology, Cantonal Hospital Sanct Gallen, Switzerland; Cleveland Clinic, UNITED STATES

## Abstract

**Background:**

Diagnosis of inflammatory bowel disease (IBD) in primary care (PC) is challenging and associated with a considerable diagnostic delay. Using a calprotectin test for any PC patient with abdominal complaints would cause significant costs. The 8-item-questionnaire CalproQuest was developed to increase the pre-test probability for a positive Calprotectin. It is a feasible instrument to assess IBD in PC, but has not yet been evaluated in clinical routine. This study, therefore, aimed to validate whether the CalproQuest increases pretest-probability for a positive fecal Calprotectin.

**Methods:**

Prospective diagnostic trial. The CalproQuest consists of 4 major and 4 minor questions suggestive for IBD. It is considered positive if ≥ 2 major or 1 major and 2 minor criteria are positive. Primary outcome: Sensitivity and specificity of the CalproQuest for Calprotectin levels ≥ 50 μg/g and for positive IBD diagnosis among patients referred to endoscopic evaluation at secondary care level. Secondary finding: Patient-reported diagnostic delay.

**Results:**

156 patients from 7 study centers had a complete CalproQuest and fecal Calprotectin test. The sensitivity and specificity of CalproQuest for Calprotectin ≥ 50 μg/g was 36% and 57%. The sensitivity and specificity of the CalproQuest for positive IBD diagnosis was 37% and 67%. The diagnostic delay was 61 months (SD 125.2).

**Conclusion:**

In this prospective diagnostic study, the sensitivity and specificity of CalproQuest for Calprotectin levels ≥ 50 μg/g and positive IBD diagnosis were poor. Additional prospective studies concerning the ideal cut-off values, validity and cost-effectiveness of a combined use with the Calprotectin test in the PC setting are necessary.

## Introduction

The estimated prevalence of inflammatory bowel disease (IBD), consisting of Crohn’s Disease (CD), ulcerative colitis (UC) and indeterminate colitis (IC) [1], is 0.2% in the western world [2]. Hence, it is not surprising that physicians are often faced with the diagnostic challenge to differentiate patients with IBD from functional gastrointestinal disorders, namely irritable bowel syndrome (IBS), based on its much higher prevalence estimated at 10–15% [3]. These challenges are reflected in the considerable diagnostic delay of IBD [1], associated with a significantly increased risk of morbidity and mortality [4]. Although IBS-like symptoms are frequently reported in patients before IBD is diagnosed [5], it is not useful that every patient undergoes an invasive endoscopic examination. As an intermediary diagnostic approach the fecal Calprotectin has been shown to reflect intestinal inflammation in patients with known IBD [6–12] and to differentiate IBD from IBS, depending on the cut-off value used. [13–16] Nevertheless, the Calprotectin test is not routinely performed in primary care (PC). The pre-test probability would be very low if a Calprotectin test would be used in all patients with abdominal complaints [17, 18]. A further problem is the low specificity of the test (many possible differential diagnoses for a positive Calprotectin test besides IBD such as esophagitis, gastritis, gastric ulcers, celiac disease, benign or malignant polyps and cancer, infectious gastroenteritis/colitis, diverticulitis, microscopic and ischemic colitis, NSAR enteropathy, lactose intolerance) and relatively high costs (currently about 60 Euros). Systematic data regarding the use of fecal Calprotectin test especially in PC are lacking.

To increase pretest-probability for a positive test result of the fecal Calprotectin test aiming at diagnosing IBD in an earlier stage, we developed an 8-item-questionnaire: the CalproQuest [19]. The CalproQuest has been shown to be a feasible instrument for the assessment of IBD in PC, but has not yet been evaluated clinically [20]. This study aims to prospectively validate the CalproQuest concerning sensitivity and specificity for positive fecal Calprotectin test results and positive IBD diagnosis.

## Materials and methods

### Ethics, trial registration, informed consent

- Ethics: The study protocol was approved by the Ethics Committee of the Kanton Zurich (reference KEK-ZH-number 2013–0516).
- The study protocol conforms to the ethical guidelines of the 1975 Declaration of Helsinki as reflected in a prior approval by the institution’s human research committee.
- Trial registration number: ISRCTN66310845.
- Written informed consent was obtained from each patient included in the study.

### Study design

**Fig 1:**
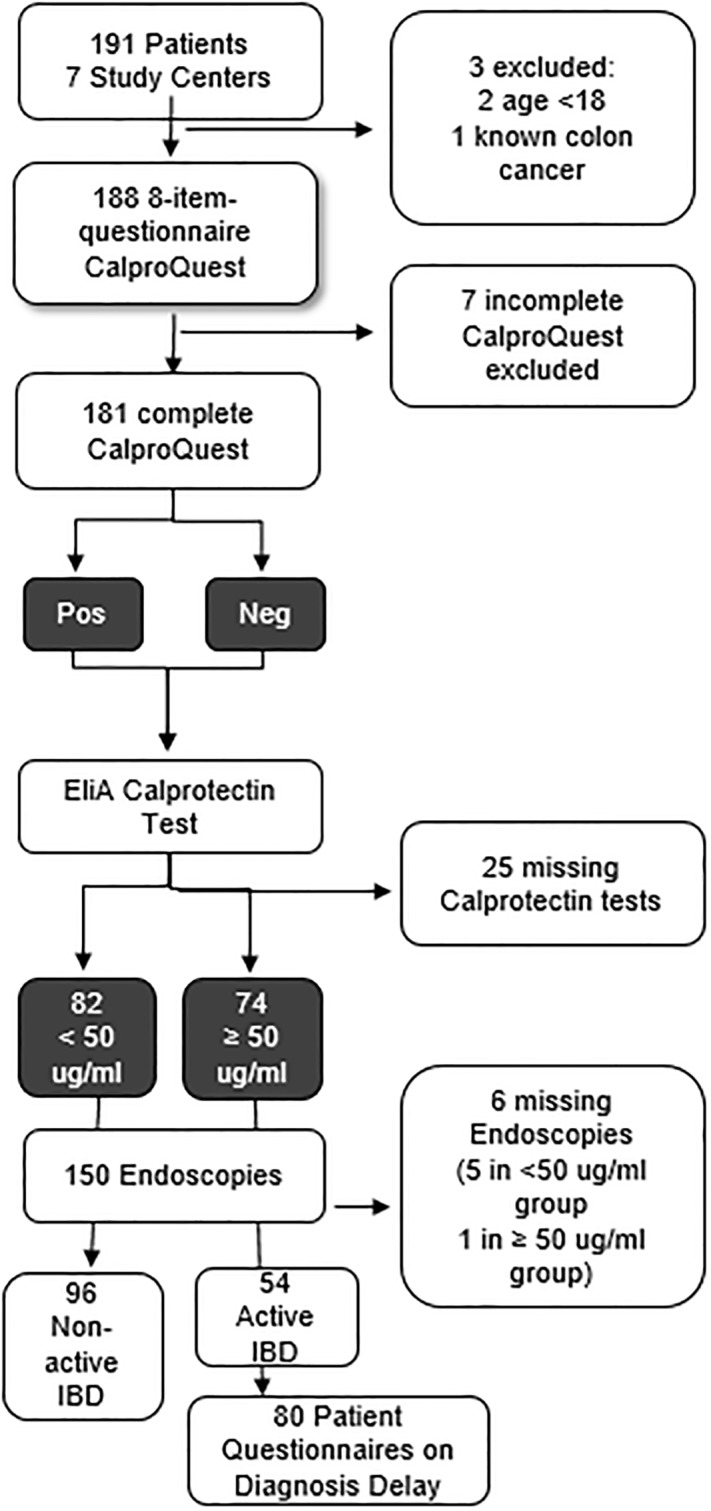
Study flow Feasibulity of CalproQuest. Neg = negative; Pos = positive; IBD = Inflammatory bowel disease.

This study is a part of the prospective diagnostic ALERT trial (V**A**lidation of an 8-item-questionnaire predictive for a positive ca**L**protectin t**E**st and **R**eal-life implemen**T**ation in primary care to reduce diagnostic delay in IBD), consisting of two independent parts A and B, conducted by gastroenterologists (GEs) (A) and general practitioners (GPs) (B). The details of the study design, including recruitment of patients and physicians, administration of patient records, informed consent, confidentiality have been published previously [19]. In part B of the ALERT Trial, the CalproQuest has been shown to be a feasible instrument for the assessment of IBD in PC [20]. Patients included in the current study were referred to GEs for endoscopic evaluation with any indication. The study design including the study flow is depicted in Fig 1.

### Inclusion and exclusion criteria

**Inclusion criteria**:

- ≥18 years
- Referral to GE for colonoscopy with any indication
- Informed consent

**Exclusion criteria**:

- Known abdominal pathologies besides known IBD, e.g. cancer

### Procedure (see also Fig 1)

- Patients referred to the GE for colonoscopy due to any reason were included into the study (besides in- and exclusion criteria)
- Patients were subjected to CalproQuest prior to endoscopy
- Patients obtained fecal samples to measure Calprotectin levels
- Colonoscopy was performed to obtain diagnosis
- Patients with an IBD diagnosis were asked to complete a questionnaire investigating duration of first onset of symptoms to IBD diagnosis (diagnostic delay)

### CalproQuest

CalproQuest is an 8-item IBD-questionnaire consisting of 4 major and 4 minor questions suggestive for IBD (Table 1). The CalproQuest has been shown to be a feasible instrument for the assessment of IBD in PC [20]. The original questionnaire used in our study (German language) can be seen in supporting information (S1 File). The CalproQuest is considered positive, if ≥ 2 major criteria or 1 major criterion and 2 minor criteria are answered positively. We assumed that a positive CalproQuest result might predict calprotectin levels ≥ 50 μg/g. Calprotectin levels above 50 μg/g are indicative for active intestinal inflammation and call for further endoscopic examination.

**Table 1:** CalproQuest (8-item inflammatory bowel disease questionnaire).

Type	Criteria	Yes (1)	No (0)	Comment
Major	Does the patient suffer from abdominal pain at least 3 times a week for at least 4 weeks?			
	Does the patient suffer from diarrhea (more than 3 bowel movements daily) for 7 consecutive days?			
	Does the patient have diarrhea at night-time/Does the patient awake from sleep because of abdominal pain or diarrhea?			
	Does the patient report bloody stool?			
Minor	Does the patient report mucus in stool for more than 4 weeks?			
	Does the patient report unwanted weight loss (5% of normal body weight over 6 months)?			
	Does the patient present with fever or report fever over the last 4 weeks (Temperature > 38°C)?			
	Does the patient report fatigue over the last 4 weeks?			

### Fecal calprotectin

Fecal calprotectin levels were measured at the University Hospital Zurich. Specimens from other study centers were sent to the laboratory by mail. The Calprotectin test is called EliA Calprotectin (Thermo Fisher Scientific, Uppsala, Sweden) and uses the FEIA method (fluorescence enzyme immunoassay) on a fully automated system called Phadia 100 (Thermo Fisher Scientific, Uppsala, Sweden). The EliA Calprotectin Wells are coated with monoclonal antibodies to calprotectin. If present in the patient’s specimen, calprotectin binds to the coated antibodies. After washing away non-bound components, enzyme-labeled antibodies against human calprotectin (EliACalprotectin Conjugate) are added to form a calprotectin-conjugate complex. After incubation, non-bound conjugate is washed away and the bound complex is incubated with a Development Solution. After stopping the reaction, the fluorescence in the reaction mixture is measured. The higher the response value, the more calprotectin is present in the specimen. To evaluate test results, the response for patient samples is compared directly to the response for calibrators.

### Diagnostic delay

Three time intervals of diagnostic delay were assessed retrospectively in a patient questionnaire (S2 File in German language): Interval 1: Time from first IBD-related symptoms to first consultation with a physician. Interval 2: Time from the first physician visit to referral to GE: Interval 3: Time from first IBD symptoms to IBD diagnosis (interval 1+2): This interval is defined as diagnostic delay and describes time span from first symptoms to IBD diagnosis.

### Primary and secondary outcomes

**Primary outcomes**:

- Sensitivity and specificity of CalproQuest for a positive Calprotectin test result ≥ 50 μg/g feces
- Sensitivity and specificity of CalproQuest for a positive Calprotectin test result ≥ 50 μg/g feces and positive IBD-diagnosis.

**Secondary finding**:

- Patient-reported diagnostic delay

### Statistical analysis

A data set was considered complete if a patient completed both the CalproQuest and the Calprotectin test. The sensitivity and specificity calculation of CalproQuest is based on confidence intervals. P<0.05 is considered statistically significant. The sample size was calculated according to Flahault et al. [21]. Assuming a 0.05 two-sided significance level, n = 162 would have 90% power to detect a sensitivity and specificity of 90% of CalproQuest for a calprotectin level ≥ 50 μg/g feces, or for a calprotectin level ≥ 50 μg/g feces and a positive IBD diagnosis. For the purpose of this calculation, the expected sensitivity and specificity are 90% with a lower acceptable limit of sensitivity of 70%. Assumed prevalence of IBD within the sample was 20%. A p<0.05 is considered statistically significant. Statistical analysis was performed with R (R version 3.3.2) [22].

## Results

### Population

Recruitment of GEs started in October 2014 and ended after completion of the necessary dataset in January 2017. Recruitment was undertaken by means of information events as well as mailings and personal contacts of the involved team. Therefore, no actual non-responder list was compiled. The study flow can be appreciated in Fig 1.

From the 191 eligible patients in 7 study centers, 188 remained for analysis meeting the inclusion and exclusion criteria, 156 had a complete CalproQuest as well as a Calprotectin test (Fig 1 and Table 2). The centers recruited between 3 and 19 patients. The details of the study population are listed in Table 2. 150 endoscopic results were available for analysis, of which 80 hat an IBD diagnosis (54 active, 24 in remission). 21 endoscopies showed other diagnoses (e.g. diverticulitis, diverticulosis, etc.). 49 endoscopies showed no pathological findings.

**Table 2:** Baseline characteristics of the study population.

	level	frequency	percent
CalproQuest (n = 156)	negative	94	60.3
	positive	62	39.7
Sex (n = 155)	female	75	48.4
	male	80	51.6
First endoscopy (n = 150)	No	86	57.3
	yes	64	42.7
Follow up endoscopy (n = 150)	No	64	42.7
	yes	86	57.3
Active Crohn’s disease (n = 150)	No	126	84
	yes	24	16
Active colitis ulcerosa (n = 150)	0	126	84
	1	24	16
Active indeterminate inflammatory bowel disease (n = 150)	0	144	96
	1	6	4
Active inflammatory bowel disease (Crohn’s or colitis ulcerosa or indeterminate) (n = 150)	0	96	64
	1	54	36
Crohn in remission (n = 150)	0	137	91.3
	1	13	8.7
Colitis ulcerosa in remission (n = 150)	0	137	91.3
	1	13	8.7
Other diagnoses (n = 150)	Diverticulitis	2	2.9
	Diverticulosis	6	8.6
	Diverticulosis and polyp	3	4.3
	Hemorrhoids	2	2.9
	Microscopic colitis	2	2.9
	No findings	49	70
	Polyp	6	8.6
Medication (n = 132)	O	71	59.7
	C	2	1.7
	C, O	7	5.9
	C, N	1	0.8
	C, N, O	2	1.7
	N	2	1.7
	N, O	10	8.4
	S	3	2.5
	S, O	15	12.6
	S, C, O	3	2.5
	S, C, N, O	1	0.8
	S, N, O	2	1.7

n = available data, C = contraceptives, N = non-steroidal anti-inflammatory rheumatics, S = steroids, O = other.

### Primary outcome: Validation of CalproQuest

**Fig 2:**
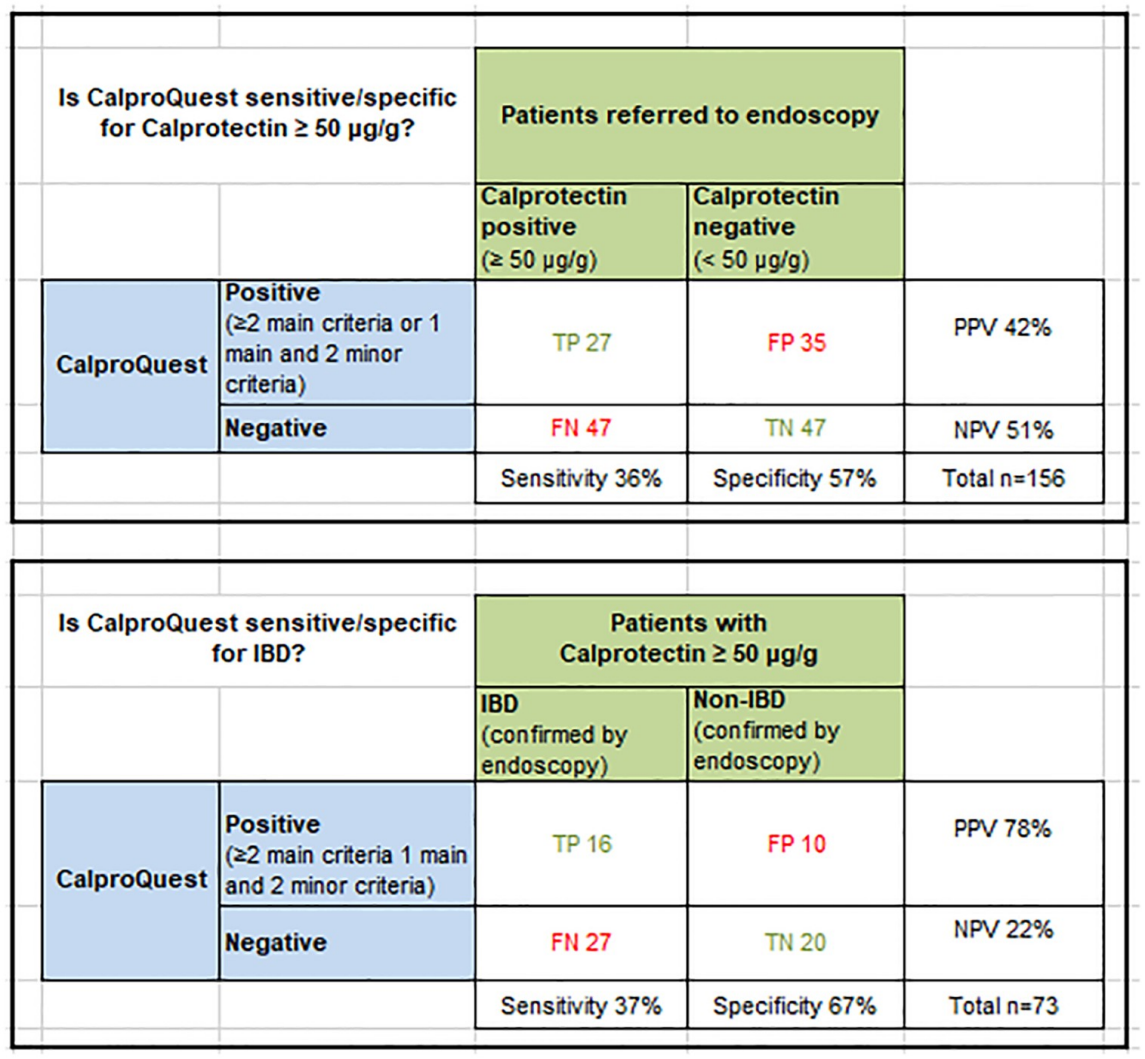
Sensitivity, Specifity and predictive values of the CalproQuest. TP = true positive, TN = true negative, FP = false positive, FN = false negative, PPV = Positive Predictive Value = TP/(TP+FP), NPV = Negative Predictive Value = TN/(FN+TN), Sensitivity = TP/ (TP+FN), Specificity = TN/(FP+TN), IBD = Inflammatory bowel disease.

The sensitivity and specificity of CalproQuest for fecal Calprotectin levels ≥ 50 μg/g was 36% and 57% (n = 156). Positive IBD diagnosis was defined as endoscopic diagnosis of active CD or UC or IC (n = 54). The sensitivity and specificity of the CalproQuest for positive IBD diagnosis was 37% and 67% (Fig 2).

**Fig 3:**
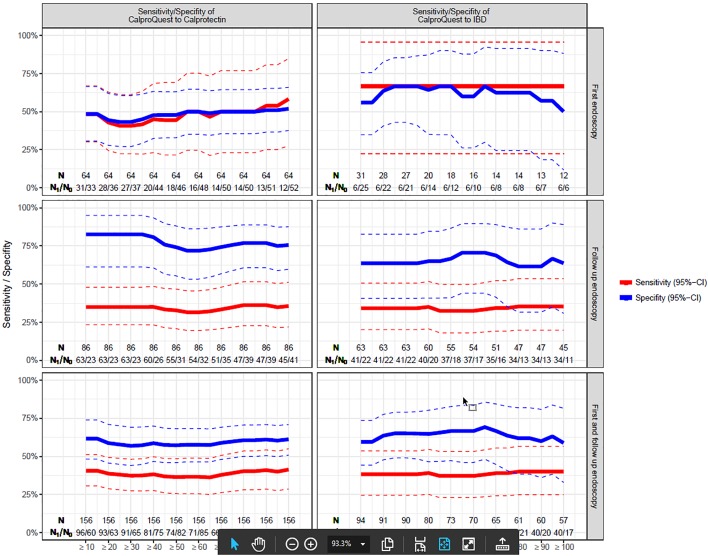
Sensitivity and Specificity of the CalproQuest depending on Calprotectin levels and on first or follow up endoscopy. No association was found between the sensitivity and specifity of the CalproQuest with different Calprotectin levels or with the endoscopy being performed for the first time or as a follow up endoscopy.

Fig 3 shows the sensitivity and specificity of CalproQuest depending on different Calprotectin levels and on whether the diagnosis of IBD was assessed in a first or follow up endoscopy.

### Secondary finding: Patient-reported diagnostic delay

The mean time from first IBD related symptoms to first consultation with a physician (Interval 1) was 6 months (SD 16.3). The mean reported time from the first physician visit to referral to a gastroenterologist (Interval 2) was 19 months (SD 58.6). The diagnostic delay (= time from first IBD symptoms to IBD diagnosis (Interval 3 = Interval 1+2)) was 61 months (SD 125.2).

## Discussion

In this prospective diagnostic study, the sensitivity and specificity of CalproQuest, an 8-item IBD-questionnaire consisting of 4 major and 4 minor questions, for fecal Calprotectin levels ≥ 50 μg/g as well as positive IBD diagnosis were poor.

The CalproQuest has been shown to be a feasible instrument for the assessment of IBD in PC care [20]. In the current prospective diagnostic study, the CalproQuest is for the first time evaluated clinically. The question arises why the results turned out poor. In current literature, very few studies exist evaluating the use of questionnaires to aid GP’s ruling out IBD, hence it is difficult to compare our results. Danese et al. [23] published a 21-item questionnaire, which was developed by means of a systematic literature review in which CD specialists identified “red flags”, i.e. symptoms or signs suggestive of CD. However, this questionnaire was not yet tested for feasibility in PC and has not yet been prospectively validated. As Holtman et al. [24] have stated correctly, low prevalence of IBD and lack of uniform reference standards in PC induce methodological challenges to investigate the diagnostic accuracy of a test. In Switzerland only about 7% of patients consult their GP due to gastrointestinal complaints [25], of which only a minority (0.2%) is diagnosed with IBD [2], whereas the prevalence of IBS is estimated at 10–15% [3]. In order to achieve the necessary power to estimate the sensitivity and specifity of the CalproQuest in the PC setting, more than 2000 patients would have had to be recruited, due to the low prevalence of gastrointestinal complaints in PC. In the current study, we therefore decided to estimate the diagnostic performance of the CalproQuest at secondary care, i.e. among the GEs. By recruiting 156 patients with a complete data set (complete CalproQuest and Calprotectin), we nearly achieved the targeted number of 163 patients, based on the power calculation. Taking into consideration that the active IBD prevalence in our study population was 34.6%, i.e. substantially higher than the 20% as estimated in the power calculation [19], the power was actually more than achieved. The mediocre diagnostic accuracy of the CalproQuest for a positive Calprotectin therefore represents a true finding and cannot be explained by the study design. A possible explanation for the poor results might lie in the diagnostic accuracy of the fecal calprotectin test itself and the low cut-off value of 50 μg/g chosen in our study. Although testing for fecal calprotectin has been shown to be a helpful diagnostic tool for IBD in tertiary care and especially as a follow-up parameter (13–16), it remains unclear whether its widespread use for diagnostic rather than follow-up purposes in primary and secondary care is appropriate. The fecal calprotectin test was not validated in the low prevalence setting of PC [26] and has diverse differential diagnoses for a positive result besides IBD, which renders its utility even more unclear in a low prevalence setting. Recently published studies in the PC setting have shown a doubtful benefit of the calprotectin test in diagnosing IBD in PC [27], whereas other non-invasive markers, such as the fecal immunochemical test (FIT), showed far better results in detecting colorectal cancer, high-risk adenomas and IBD [28], [29]. Also in the pediatric PC setting recent findings indicate that the fecal calprotectin test may not be as helpful as assumed from specialist care: A positive fecal calprotectin result in children with chronic gastrointestinal symptoms was not likely to be indicative of IBD [30]. It seems reasonable that the cut-off value has to be reconsidered for this low prevalence setting, in order to increase utility of a positive calprotectin test (positive predictive value) [27], as suggested in studies among patients with an intermediate raised fecal calprotectin in a ‘real-world’ setting [31–33]. A negative calprotectin tests seems to have certain utility in ruling out IBD in PC [34, 35]. In addition, the questionable cost-effectiveness of a widespread use in a patient population with unspecific complaints has to be taken into consideration.

### Strengths and limitations

A clear strength of this study is its clinical relevance: as a secondary finding, the diagnostic delay was confirmed to be substantial and hence comparable with previous studies. [1, 4] Since this substantial diagnostic delay is clearly associated with an increased risk of morbidity and mortality [4], studies like this one tackling the issue how to timely diagnose these patients are of utmost importance.

Certain limitations have to be taken into consideration: As mentioned above, in order to achieve the necessary power to estimate sensitivity and specificity of the CalproQuest in the GP setting, more than 2000 patients would have had to be recruited, due to this low prevalence of gastrointestinal complaints in PC. In the current study, we therefore chose to estimate the diagnostic performance of the CalproQuest at secondary care level, i.e. among GEs. In addition, there is evidence for great variability in the concentrations of calprotectin in stool samples collected during a single day with increasing variability of concentrations in longer storage periods [36]. Since our study protocol did not predefine a specific storage time, our study results could have been negatively influenced. Since 15% the patients in the study took non-steroidal anti rheumatics, which are known to increase fecal Calprotectin levels, our findings could have been influenced [37]. Since the exact and cumulative dosages of medication intake was not known, further sub-analyses are not possible to correct for this confounding element.

In summary, the poor predictive power for the CalproQuest might possibly be improved to reach a major diagnostic power by means of the following measures: more specific instructions concerning stool sampling procedure, especially concerning storage time, the use of another potentially better fecal test, than the Calprotectin, such as the fecal immunochemical test (FIT), the use of a higher cut of level of the Calprotectin, and finally testing in the PC rather the secondary care setting with a larger sample size.

## Conclusion

In this prospective diagnostic study, the sensitivity and specificity of CalproQuest for fecal Calprotectin levels ≥ 50 μg/g as well as positive IBD diagnosis were poor. Additional prospective studies concerning the ideal cut-off values, validity and cost-effectiveness of a combined use with the Calprotectin test in the PC setting are necessary.

## Supporting information

S1 File CalproQuest. Questionnaire for the early detection of inflammatory bowel disease (German language).
(DOCX)

S2 File Diagnostic delay. Questionnaire on patient history for patients suffering from Cohn’s disease or colitis ulcerosa (German language).
(DOCX)

S3 File Study protocol Ethics. Ethics consent.
(PDF)

S1 Table TREND Checklist. Transparent Reporting of Evaluations with Nonrandomized Designs.
(DOCX)

S2 Table Raw data. Raw data.
(XLSX)

S1 Fig CONSORT flow Diagram. Consolidated Standards of Reporting Trials.
(DOC)

## Abbreviations

CalproQuest: 8-item-questionnaire to increase pretest-probability for a positive test result of the fecal Calprotectin test
CD: Crohn’s Disease
GP: General Practitioner
IBD: Inflammatory Bowel Disease
IBS: Irritable bowel syndrome
IC: Indeterminate colitis
UC: Ulcerative colitis
