# Supplementary material for: Can the CalproQuest predict a positive Calprotectin test? A prospective diagnostic study
Source: PLoS One. 2019 Nov 21;14(11):e0224961. doi: 10.1371/journal.pone.0224961 (PMC6872045; doi:10.1371/journal.pone.0224961)
Supplement: S1 File — Questionnaire for the early detection of inflammatory bowel disease (German language). (DOCX) [file pone.0224961.s001.docx]

**CalproQuest**

**Arzt-Patienten Fragebogen für die frühe Detektion von chronisch Entzündlichen Darmerkrankungen (CED)**

**Fragen zur Verdachtsabklärung von chronisch entzündlichen Darmerkrankungen:**

| **Kriterium** | **Frage** | **Ja**  **(1)** | **Nein**  **(0)** | **Kommentar** |
| --- | --- | --- | --- | --- |
| Haupt | Leidet der Patient seit mindestens 4 Wochen an Bauchschmerzen, welche mindestens an 3 Tagen pro Woche auftreten? |  |  |  |
|  | Zeigt der Patient Durchfall^1^ über mehr als an 7 aufeinanderfolgenden Tagen? |  |  |  |
|  | Hat der Patient nächtlichen Durchfall? |  |  |  |
|  | Berichtet der Patient über Blut^2^ im Stuhl?^$^ |  |  |  |
| **Total Hauptkriterien** | |  |  |  |
| Neben | Berichtet der Patient über Schleim^3^ im Stuhl über mehr als 4 Wochen? |  |  |  |
|  | Berichtet der Patient über ungewollten Gewichtsverlust^4^? |  |  |  |
|  | Berichtet der Patient über Fieber^5^ in den letzten 4 Wochen oder hat er akut Fieber? |  |  |  |
|  | Berichtet der Patient über Müdigkeit^6^ in den letzten 4 Wochen? |  |  |  |
| **Total Nebenkriterien** | |  |  |  |

$: immer Differentialdiagnose Kolon-Karzinom bedenken

CalproQuest wird als positiv betrachtet, wenn ≥ 2 Hauptkriterien oder 1 Hauptkriterium und ≥ 2 Nebenkriterien positiv beantwortet wurden.

**Zusätzliche Fragen zu dem/der untersuchten Patienten/Patientin:**

Jahrgang: ___________________ Geschlecht: □ männlich □ weiblich

Aktuelle Medikation: ___________________ Raucher: □ ja □ nein

___________________

___________________

___________________

**CalproQuest**

**Arzt-Patienten Fragebogen für die frühe Detektion von chronisch Entzündlichen Darmerkrankungen (CED)**

**Definitionen:**

| 1 Durchfall | Mehr als 3 Stuhlgänge pro Tag (oder 3 Stuhlgänge mehr als normal, falls drei Stuhlgänge pro Tag der Normalfall ist).  Konsistenz des Stuhls ungeformt oder kaum geformt, mit sichtbar wässerigem Anteil. |
| --- | --- |
| 2 Blut im Stuhl | Erkennbar durch blutige Anteile im Stuhl, welche das Wasser in der Toilette rot/rötlich färben. Wenn das Blut nur beim Saubermachen auf dem Toilettenpapier sichtbar wird, ist eine Hämorrhoide abzuklären. Ist auch einmalig als Alarmzeichen zu interpretieren, immer Kolonkarzinom als Differentialdiagnose bedenken. |
| 3 Schleim im Stuhl | Erkennbar durch weisse schleimige Anteile, welche im Stuhl sichtbar sind.  Da der Schleim nicht immer klar zu sehen ist, ist eine rapportierte Häufigkeit von 1-2x/Woche ausreichend. Patienten erkennen den Schleim oft auch am schmierigen Gefühl beim Saubermachen. |
| 4 Gewichtsverlust | Definiert als einen ungewollten Gewichtsverlust von 5% über die letzten 6 Monate. Wenn sich der Patient nicht wiegt: 5% entsprechen ca. einer halben Kleidergrösse und die Nachfrage nach zu lockeren Hosen kann hilfreich sein. Falls unklar, aber eher ja, sollte das Kriterium positiv bewertet werden. |
| 5 Fieber | Wird mit mehr als 38°C definiert. Fiebriges Gefühl von Patienten, welches klar nicht dem normalen Erleben zugeordnet werden kann, kann für die Verwendung in diesem Fragebogen als Fieber interpretiert werden. |
| 6 Müdigkeit | Wird definiert mit Abgeschlagenheit und verminderter Antriebsfähigkeit, welche auch durch genügend Schlaf nicht kuriert werden kann. Typisch ist auch ein bleiernes Gefühl im Körper beim Aufstehen, welches nicht dem Normalzustand entspricht. |
