# Supplementary material for: Can the CalproQuest predict a positive Calprotectin test? A prospective diagnostic study
Source: PLoS One. 2019 Nov 21;14(11):e0224961. doi: 10.1371/journal.pone.0224961 (PMC6872045; doi:10.1371/journal.pone.0224961)
Supplement: S2 File — Questionnaire on patient history for patients suffering from Cohn’s disease or colitis ulcerosa (German language). (DOCX) [file pone.0224961.s002.docx]

**Diagnostic Delay**

Sie leiden an Morbus Crohn oder Colitis ulcerosa. Bitte nehmen Sie sich fünf Minuten Zeit für ein paar Fragen zu Ihrer Krankheitsgeschichte.

1. Woran leiden Sie?
   - Morbus Crohn
   - Colitis ulcerosa
2. Welchen Arzt haben Sie als erstes aufgesucht, als Sie die Beschwerden bemerkt haben?
   - Hausarzt
   - Spezialist (Gastroenterologe)
3. Nachdem Sie die ersten Beschwerden und Symptome bemerkt haben, wie lange haben Sie zugewartet, um Ihren Hausarzt zu besuchen?
   - _______________________Tage
   - _______________________Wochen
   - _______________________Monate
   - _______________________Jahre
4. Wie lange haben Sie an Krankheitsbeschwerden gelitten, bevor die Erkrankung diagnostiziert wurde?
   - _______________________Tage
   - _______________________Wochen
   - _______________________Monate
   - _______________________Jahre
5. Wie lange wurden Sie von Ihrem Hausarzt untersucht und/oder behandelt, bevor Sie zum Gastroenterologen überwiesen wurden?
   - _______________________Tage
   - _______________________Wochen
   - _______________________Monate
   - _______________________Jahre
6. Wie viel Zeit ist vergangen vom ersten Arzt-Besuch bis zur Diagnose?
   - _______________________Tage
   - _______________________Wochen
   - _______________________Monate
   - _______________________Jahre
7. Haben Sie zum Zeitpunkt der Diagnose geraucht?
   - Nein
   - Ja. Wie viel? ______________________________________
8. Haben Sie zum Zeitpunkt der Diagnose eines der folgenden Medikamente eingenommen?
   - Kontrazeptive (Empfängnisverhütung)
   - Nichtsteroidale Antirheumatika (Aspirin, Ibuprofen, Diclofenac, Mefenaminsäure, Coxibe)
   - Andere__________________________________________
