# Supplementary material for: Can the CalproQuest predict a positive Calprotectin test? A prospective diagnostic study
Source: PLoS One. 2019 Nov 21;14(11):e0224961. doi: 10.1371/journal.pone.0224961 (PMC6872045; doi:10.1371/journal.pone.0224961)
Supplement: S3 File — Ethics consent. (PDF) [file pone.0224961.s003.pdf]

ALERT Study Protocol

V**A**lidation of an 8-item-questionnaire predictive for a positive Ca**L**protectin t**E**st and **R**eal-life implemen**T**ation in primary care to reduce diagnostic delay in inflammatory bowel disease

Sponsor

IBDnet, PD. Dr. med. Stephan Vavricka

Principal investigators

Prof. Dr. med. Thomas Rosemann, PhD

Participating Center

Universitätsspital Zürich

Date and place

Zürich, 27. 5. 2014

Signature

Prof. Dr. med. Thomas Rosemann, PhD

PD Dr. med. Stephan Vavricka

Universität Zürich[UZH] UniversitätsSpital Zürich Institut für Hausarztmedizin IBDnet

Content

Background 3
Calprotectin, a S100 protein 3
Stool Calprotectin Levels as Marker of Intestinal Inflammation 3
Diagnostic delay in IBD is predictive for worse disease progression and outcomes 3
Testing calprotectin in Switzerland 4

Hypothesis and goal 5

Methods and study design 6
Study design 6
Inclusion and exclusion criteria 6
Primary and secondary outcomes 6
Procedure of the study 6
Time frame 7
CalproQuest 7
Fecal calprotectin 7
Patient questionnaire on diagnostic delay 8
Physician questionnaire on feasibility and acceptance of CalproQuest in primary care 8
Patient questionnaire on acceptance of stool sampling 9
Administration of patient records 9

Statistical analysis 10
Sample size calculation 10
Statistical data evaluation 10

References 11

Addendum 13
Study design 13
CalproQuest (8-item IBD questionnaire) 14
Patient questionnaire: Diagnostic Delay 15
Physician questionnaire: Feasibility and acceptance of CalproQuest in primary care 16
Patient questionnaire: Acceptance of stool sampling 17

Background

Calprotectin, a S100 protein

Calprotectin is a complex of two calcium-binding proteins that belong to the S100 protein family[1]. It is abundant in the cytosolic fraction of neutrophils. High levels of calprotectin have been found in extracellular fluid during various inflammatory conditions, such as rheumatoid arthritis, cystic fibrosis and abscesses. Calprotectin released from neutrophils has growth-inhibitory and apoptosis-inducing activities against various cell types including tumor cells and normal fibroblasts[1]. This suggests that calprotectin has regulatory activities during inflammatory processes through its effect on the survival or growth states of cells participating in the inflammatory reaction.
Furthermore, calprotectin inhibits microbial growth through competition for zinc[2].
Calprotectin has been shown to be stable in feces during storage for 7 days at room temperature, which is very important for its value in evaluating mucosal wall inflammation[3].

Stool Calprotectin Levels as Marker of Intestinal Inflammation

Fecal Calprotectin has been shown to consistently differentiate inflammatory bowel disease (IBD) from irritable bowel syndrome (IBS) because it has an excellent negative predictive value in ruling out IBD in undiagnosed, symptomatic patients[4].
Convincing studies and growing clinical experience point to an expanded role in the diagnosis and management of IBD.
A total 602 new referrals to a gastroenterology clinic who had symptoms suggestive of IBS or organic intestinal diseases underwent invasive imaging and other investigations as appropriate. Data correlated to fecal Calprotectin showed a sensitivity and specificity of Calprotectin for organic disease of 89% and 79%, respectively[5]. This indicates that fecal calprotectin provides a safe and non-invasive marker to differentiate between patients with organic and non-organic intestinal disease[6].
Fecal calprotectin levels correlate significantly with histological and endoscopic assessment of disease activity in ulcerative colitis (UC)[6-8] as well as with fecal alpha-1-antitrypsin levels and fecal excretion of 111indium-labeled white blood cells in patients with Crohn's disease (CD)[9, 10].
Schoepfer and colleagues showed that stool calprotectin levels correlate well with endoscopic indices both in ulcerative colitis and in Crohn's disease[11, 12].

Diagnostic delay in IBD is predictive for worse disease progression and outcomes

Diagnosing IBD can be a considerable challenge, especially in cases with mild clinical activity due to the overlap of symptoms with functional diseases. Indeed, symptoms similar to irritable bowel syndrome (IBS) are frequently reported in patients before IBD is diagnosed[13]. Difficulties in differentiation early IBD from IBS, especially in a primary care setting, is leading to a considerable diagnostic delay in IBD[14]. Diagnostic delay has important clinical impact, as there is increasing evidence demonstrating that treatment success is increased in early disease[15-19]. Vavricka and colleagues suggest, that diagnostic delay is sub-dived into two intervals, where interval 1 is defined as time from first symptoms to physician visit, and interval the time from first physician visit to IBD diagnosis[14]. The study by Vavricka et al suggests, that 25% of all CD and UC patients wait from first onset of symptoms more than 24 and 12 months, respectively, for their accurate IBD diagnosis[14]. Most importantly Schoepfer and colleagues recently showed, that the length of diagnostic delay is correlated with an increased risk of bowel stenosis and CD-related intestinal surgery, concluding that efforts should be undertaken to shorten the diagnostic delay[21].

Testing calprotectin in Switzerland

Most analytical laboratories in Switzerland offer calprotectin testing, which is reimbursed by health insurances.

Hypothesis and goal

Although calprotectin test are easily accessible and reimbursed in Switzerland, the diagnostic value for early detection of intestinal inflammation is not yet recognized in primary care setting, leading to a significant diagnostic delay[14].

This study pursuits two main goals A and B, which are investigated independently:

A. Retrospective validation and evaluation of sensitivity and specificity of an 8-item IBD-questionnaire (CalproQuest; see addendum) for 1) a positive Calprotectin test result $\geq$ 50 µg/ml feces and for 2) a positive Calprotectin test result $\geq$ 50 µg/ml feces and positive IBD-diagnosis, respectively.

B. Prospective implementation of CalproQuest in primary care to investigate feasibility in daily practice.

Methods and study design

Study design

This study is a mono-centric diagnostic observational trial. The study consists of two independent and consecutive parts A and B, conducted by gastroenterologists (A) and general practitioners (B), respectively.
Patients included in part A of the study are referred to the gastroenterologist for endoscopic evaluation for any reason. Patients included in part B of the study present at their family doctor because of on-going unspecific gastrointestinal symptoms (abdominal pain, bloating, stool irregularities, chronic diarrhea) for at least two weeks.
The study design and procedure are summarized in the addendum (see study design).

Inclusion and exclusion criteria

Patients will be eligible if they

- Are ≥18 years old
- Are referred to their gastroenterologist for any endoscopic examination (Part A)
- Visit their family doctor because of on-going unspecific gastrointestinal symptoms (abdominal pain, bloating, stool irregularities, chronic diarrhea) for at least two weeks (Part B)
- Underwent no further diagnostic procedures (endoscopy) for the current episode

Patients will not be eligible, if they

- Are younger than 18 years
- Have known abdominal pathologies
- Had previous abdominal surgeries
- Have been treated with steroids (topical and/or oral) and/or aminosalicylates within 30 days prior inclusion into this study
- Underwent endoscopic examination within 3 years prior screening

Primary and secondary outcomes

Primary goals:

A. 1. Sensitivity and specificity of CalproQuest for a positive Calprotectin test result ≥ 50 µg/ml feces

   2. Sensitivity and specificity of CalproQuest for a positive Calprotectin test result ≥ 50 µg/ml feces and positive IBD-diagnosis.

B. Feasibility of CalproQuest in daily primary care practice.

Secondary goals:

A. Patient-reported diagnostic delay.
B. Patient acceptance of stool sampling.

Procedure of the study

In brief, the study will be divided in two independent parts A and B, conducted by gastroenterologists (A) and general practitioners (B), respectively. Patient data will be encoded.

A. Investigation of the sensitivity and specificity of CalproQuest for stool Calprotectin levels ≥ 50 µg/ml feces and for positive IBD diagnosis
162 patients referred to the gastroenterologist for endoscopic examination are subjected to CalproQuest and Calprotectin stool testing prior endoscopy, if all inclusion criteria are met and informed patient consent is obtained. At baseline T0, patients will be subjected to CalproQuest. Subsequently, at T1 fecal samples will be obtained to measure calprotectin levels. The patients themselves will perform collection of the fecal specimens. The fecal specimens from outpatients will be shipped to the laboratory at the University Hospital Zurich by mail. After measurement, fecal samples will be disposed according to current guidelines. At T2, endoscopic examination will be performed to obtain a diagnosis. Eventually, patients diagnosed with IBD will be asked to complete a questionnaire at T3 investigating duration of first onset of symptoms to IBD diagnosis (diagnostic delay).

B. Investigation of feasibility of CalproQuest in daily primary care practice
80 patients with on-going unspecific gastrointestinal symptoms (abdominal pain, bloating, stool irregularities, chronic diarrhea) for more than two weeks presenting at the general practitioner (n=27) will be included into the study if all inclusion criteria are met and informed patient consent is obtained.
At baseline T0, patients will be subjected to CalproQuest. Subsequently, at T1 fecal samples will be obtained to measure calprotectin levels. The patients themselves will perform collection of the fecal specimens. The fecal specimens from outpatients will be shipped to the laboratory at the University Hospital Zurich by mail. After measurement, fecal samples will be disposed according to current guidelines. According to the current standard of care patients with calprotectin levels ≥ 50 µg/ml will be referred to a gastroenterologist for endoscopic examination at T2; results of the endoscopy are communicated back to the general practitioner. Eventually, patients will be asked at T3 to complete a questionnaire on acceptance of stool sampling, and physicians will complete the questionnaire on feasibility of CalproQuest in daily practice.

Time frame

The study is intended to last 14 months, from which the recruitment time will be 12 months and the intervention 2 months.

CalproQuest

CalproQuest is an 8-item IBD-questionnaire consisting of 4 main and 4 secondary questions specific for IBD (see addendum). CalproQuest was pre-validated by IBD-experts trough an international Delphi-process. CalproQuest is considered positive, if ≥ 2 main criteria are answered positively or 1 main criterion and 2 secondary criteria are answered positively.
We assume that a positive CalproQuest result may predict Calprotectin levels ≥ 50 µg/ml. Calprotectin levels above 50 µg/ml are indicative for on-going intestinal inflammation and call for further endoscopic examination.

Fecal calprotectin

Fecal Calprotectin levels will be measured at the University Hospital Zurich by a novel ELISA-based calprotectin test named EliA Caprotectin (Thermo Scientific, for product description see http://www.phadia.com/PageFiles/29347/Product%20information%20EliA%20Calprotectin.pdf).

Patient questionnaire on diagnostic delay

Three relevant time intervals of diagnostic delay will be assessed in a patient questionnaire (see addendum). The time intervals are defined as follows:

1. Interval 1: Time from first IBD symptoms to consultation with the general practitioner: This interval represents the time span between the first manifestations of IBD-related symptoms (patient-reported) and a consultation with the family physician specifically due to these IBD-related complaints. The length of this period is mainly dependent on the patient herself/himself.
2. Interval 2: Time from family physician visit to referral to a gastroenterologist: This represents the time span between the IBD symptom-related consultation of the family physician and the time of referral to a gastroenterologist for further examination. The length of this period is mainly dependent on the treating family physicians.
3. Interval 3: Time from first IBD symptoms to IBD diagnosis (interval 1+2): This interval is calculated by the addition of interval 1 and 2 and is defined as diagnostic delay. Diagnostic delay is defined as the time span (in weeks) from first symptoms to IBD diagnosis.

The following items in the patient questionnaires are assessed for the purpose of this study: ‘’Before the IBD diagnosis, how long did you experience symptoms that are now attributed to IBD?’’ and ‘’How long was the time interval between first symptoms and the first visit to your family physician?’’ and “How long were you treated by your family physician before referral to a gastroenterologist?” and ‘’What was the time span from the first physician visit (due to these complaints) until IBD diagnosis was established?’’ Additionally, patients will answer questions regarding smoking habits, intake of nonsteroidal anti-inflammatory drugs (NSAIDs), or oral contraception at the time of diagnosis.

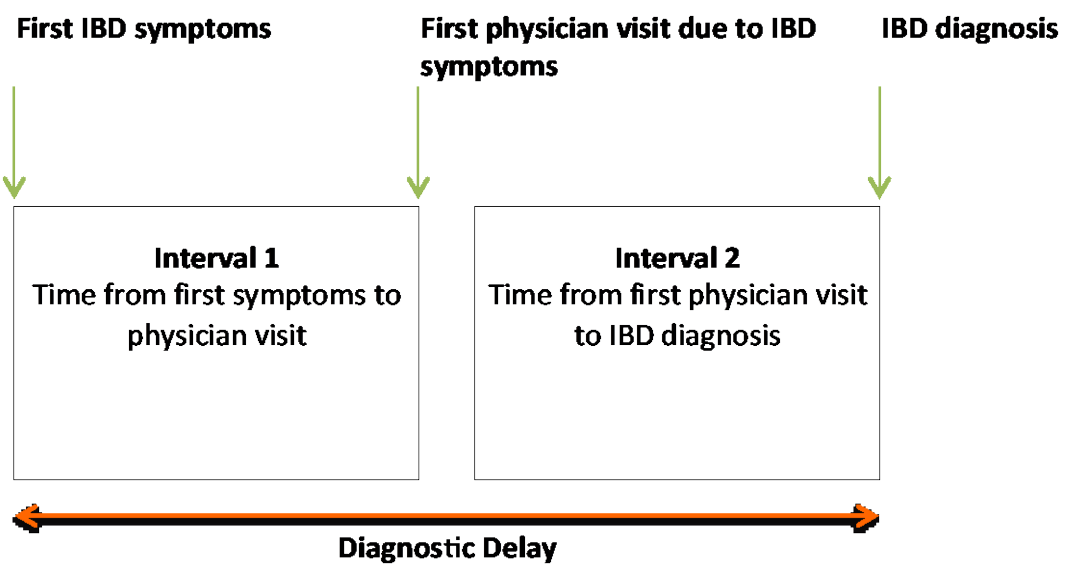

Physician questionnaire on feasibility and acceptance of CalproQuest in primary care

Goal of the feasibility questionnaire is to investigate feasibility and acceptance of CalproQuest in daily primary care practice (for questionnaire: see addendum).
The questionnaire is based on an even-point Likert scale consisting of seven Likert items.

Patient questionnaire on acceptance of stool sampling

Goal of the acceptance questionnaire is to investigate patients' physical and mental ability to handle stool sampling at home (for questionnaire: see addendum).
The questionnaire is based on an even-point Likert scale consisting of four Likert items.

Administration of patient records

Physicians will be supplied with a master data list providing patient codes that can be assigned to the patient. All documents containing patient data will carry the respective patient code assigned by the physician. Encoded documents will be sent to the Institute für Hausarztmedizin, Universitätsspital Zürich, and stored for 10 years. Only physicians have access to the patient codes.

Statistical analysis

Sample size calculation

Sample size was calculated according to Flahault et al.[22]. Assuming a 0.05 two-sided significance level, n=162 would have 90 % power to detect a sensitivity and specificity of 90% of CalproQuest for a calprotectin level ≥ 50 µg/ml feces, or for a calprotectin level ≥ 50 µg/ml feces and a positive IBD diagnosis. For the purpose of this this calculation, expected sensitivity and specificity are 90% with a lower acceptable limit of sensitivity of 70%. Assumed prevalence of IBD within the sample is 20%. A p<0.05 is considered statistically significant.

Statistical data evaluation

We provide sensitivity and specificity calculation of CalproQuest based on confidence intervals.

**Is CalproQuest sensitive/specific for Calpro ≥ 50 ug/ml?**		**Patients referred to GE for endoscopic examination**		
		**Calpro positive** (CalproELIA ≥ 50ug/ml)	**Calpro negative** (CalproELIA ≤ 50ug/ml)	
**CalproQuest**	**Positive** (≥2 main criteria OR 1 main and 2 secondary criteria)	TP	FP	**Positive Predictive Value (PPV) = TP / (TP+FP)**
	**Negative**	FN	TN	**Negative Predictive Value (NPV) = TN / (FN+TN)**
		**Sensitivity = TP / (TP+FN)**	**Specificity = TN / (FP+TN)**	

**Is CalproQuest sensitive/specific for IBD?**		**Patients with CalproELIA ≥ 50ug/ml**		
		**IBD** (confirmed by endoscopy)	**Non-IBD** (confirmed by endoscopy)	
**CalproQuest**	**Positive** (≥2 main criteria OR 1 main and 2 secondary criteria)	TP	FP	**Positive Predictive Value (PPV) = TP / (TP+FP)**
	**Negative**	FN	TN	**Negative Predictive Value (NPV) = TN / (FN+TN)**
		**Sensitivity = TP / (TP+FN)**	**Specificity = TN / (FP+TN)**	

TP	true positive	TN	true negative
FP	false positive	FN	false negative

References

1. Yui S, Nakatani Y, Mikami M. Calprotectin (S100A8/S100A9), an inflammatory protein complex from neutrophils with a broad apoptosis-inducing activity. Biol Pharm Bull 2003;26:753-60.
2. Loomans HJ, Hahn BL, Li QQ, Phadnis SH, Sohnle PG. Histidine-based zinc-binding sequences and the antimicrobial activity of calprotectin. J Infect Dis 1998;177:812-4.
3. Roseth AG, Fagerhol MK, Aadland E, Schjonsby H. Assessment of the neutrophil dominating protein calprotectin in feces. A methodologic study. Scand J Gastroenterol 1992;27:793-8.
4. Konikoff MR, Denson LA. Role of fecal calprotectin as a biomarker of intestinal inflammation in inflammatory bowel disease. Inflamm Bowel Dis 2006;12:524-34.
5. Tibble JA, Sigthorsson G, Foster R, Forgacs I, Bjarnason I. Use of surrogate markers of inflammation and Rome criteria to distinguish organic from nonorganic intestinal disease. Gastroenterology 2002;123:450-60.
6. Limburg PJ, Ahlquist DA, Sandborn WJ, Mahoney DW, Devens ME, Harrington JJ, Zinsmeister AR. Fecal calprotectin levels predict colorectal inflammation among patients with chronic diarrhea referred for colonoscopy. Am J Gastroenterol 2000;95:2831-7.
7. Roseth AG, Aadland E, Jahnsen J, Raknerud N. Assessment of disease activity in ulcerative colitis by faecal calprotectin, a novel granulocyte marker protein. Digestion 1997;58:176-80
8. Costa F, Mumolo MG, Ceccarelli L, Bellini M, Romano MR, Sterpi C, Ricchiuti A, Marchi S, Bottai M. Calprotectin is a stronger predictive marker of relapse in ulcerative colitis than in Crohn's disease. Gut 2005;54:364-8.
9. Tibble J, Teahon K, Thjodleifsson B, Roseth A, Sigthorsson G, Bridger S, Foster R, Sherwood R, Fagerhol M, Bjarnason I. A simple method for assessing intestinal inflammation in Crohn's disease. Gut 2000;47:506-13.
10. Roseth AG, Schmidt PN, Fagerhol MK. Correlation between faecal excretion of indium-111-labelled granulocytes and calprotectin, a granulocyte marker protein, in patients with inflammatory bowel disease. Scand J Gastroenterol 1999;34:50-4.
11. Schoepfer AM, Beglinger C, Straumann A, Trummler M, Renzulli P, Seibold F. Ulcerative colitis: Correlation of the Rachmilewitz endoscopic activity index with fecal calprotectin, clinical activity, C-reactive protein, and blood leukocytes. Inflamm Bowel Dis 2009;15:1851-1858.
12. Schoepfer AM, Beglinger C, Straumann A, Trummler M, Vavricka SR, Bruegger LE, Seibold F. Fecal Calprotectin Correlates More Closely With the Simple Endoscopic Score for Crohn's Disease (SES-CD) than CRP, Blood Leukocytes, and the CDAI. Am J Gastroenterol 2009.
13. Bercik P, Verdu EF, Collins SM. Is irritable bowel syndrome a lowgrade inflammatory bowel disease? Gastroenterol Clin North Am. 2005;34:235–245.
14. Vavricka SR, Spigaglia SM, Rogler G, Pittet V, Michetti P, Felley C, Mottet C, Braegger CP, Rogler D, Straumann A, Bauerfeind P, Fried M, Schoepfer AM; Swiss IBD Cohort Study Group. Systematic evaluation of risk factors for diagnostic delay in inflammatory bowel disease. Inflamm Bowel Dis. 2012 Mar;18(3):496-505.
15. Markowitz J. Early inflammatory bowel disease: different treatment response to specific or all medications? Dig Dis. 2009;27:358–365.
16. Etchevers MJ, Aceituno M, Sans M. Are we giving azathioprine too late? The case for early immunomodulation in inflammatory bowel disease. World J Gastroenterol. 2008;14:5512–5518.
17. Punati J, Markowitz J, Lerer T, et al. Effect of early immunomodulator use in moderate to severe pediatric Crohn’s disease. Inflamm Bowel Dis. 2008;14:949–954.
18. Gupta N, Bostrom AG, Kirschner BS, et al. Presentation and disease course in early compared to later-onset pediatric Crohn’s disease. Am J Gastroenterol. 2008;103:2092–2098.
19. Ricart E, Garcia-Bosch O, Ordas I, et al. Are we giving biologics too late? The case for early versus late use. World J Gastroenterol. 2008;14:5523–5527.

20. Schulz KF, Altman DG, Moher D. CONSORT 2010 Statement: updated guidelines for reporting parallel group randomised trials. BMC Med 2010, 8:18.
21. Schoepfer AM, Dehlavi MA, Fournier N, Safroneeva E, Straumann A, Pittet V, Peyrin-Biroulet L, Michetti P, Rogler G, Vavricka SR; IBD Cohort Study Group. Diagnostic delay in Crohn's disease is associated with a complicated disease course and increased operation rate. Am J Gastroenterol. 2013 Nov;108(11):1744-53.
22. Flahault A, Cadilhac M, and Thomas G. Sample size calculation should be performed for design accuracy in diagnostic test studies. Journal of Clinical Epidemiology, 2005, 58(8):859-862.

Addendum

Study design

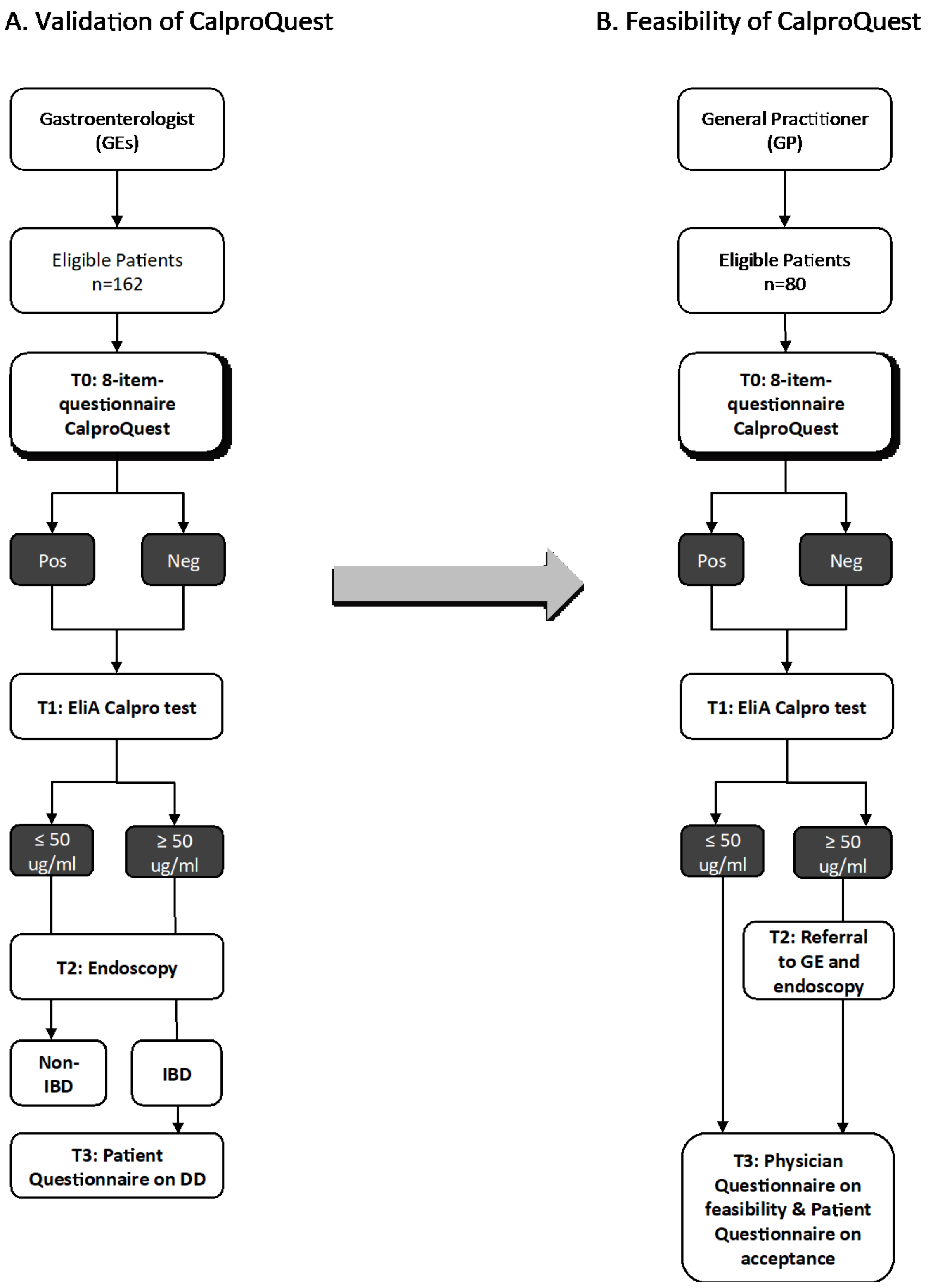

CalproQuest (8-item IBD questionnaire)

Type	Criteria	Yes (1)	No (0)	Comment
Major	Does the patient suffer from abdominal pain at least 3 times a week for at least 4 weeks?			
	Does the patient suffer from diarrhoea (more than 3 bowel movements daily) for 7 consecutive days?			
	Does the patient have diarrhoea at night-time/Does the patient awake from sleep because of abdominal pain or diarrhoea?			
	Does the patient report bloody stool?			
Minor	Does the patient report mucus in stool for more than 4 weeks?			
	Does the patient report unwanted weight loss (5% of normal body weight over 6 months)?			
	Does the patient present with fever or report fever over the last 4 weeks (Temp > 38°C)?			
	Does the patient report fatigue over the last 4 weeks?			

CalproQuest is considered positive, if ≥ 2 main criteria are answered positively or 1 main criterion and 2 secondary criteria are answered positively.

Test	Value of EliA Calprotectin test	

Patient questionnaire: Diagnostic Delay

Sie leiden an Morbus Crohn oder Colitis ulcerosa. Bitte nehmen Sie sich fünf Minuten Zeit für ein paar Fragen zu Ihrer Krankheitsgeschichte.

1. Woran leiden Sie?
   - ☐ Morbus Crohn
   - ☐ Colitis ulcerosa

2. Welchen Arzt haben Sie als erstes aufgesucht, als Sie die Beschwerden bemerkt haben?
   - ☐ Hausarzt
   - ☐ Spezialist (Gastroenterologe)

3. Nachdem Sie die ersten Beschwerden und Symptome bemerkt haben, wie lange haben Sie zugewartet, um Ihren Hausarztes zu besuchen?
   - ☐ ________________________Tage
   - ☐ ________________________Wochen
   - ☐ ________________________Monate
   - ☐ ________________________Jahre

4. Wie lange haben Sie an Krankheitsbeschwerden gelitten, bevor die Erkrankung diagnostiziert wurde?
   - ☐ ________________________Tage
   - ☐ ________________________Wochen
   - ☐ ________________________Monate
   - ☐ ________________________Jahre

5. Wie lange wurden Sie von Ihrem Hausarzt untersucht und/oder behandelt, bevor Sie zum Gastroenterologen überwiesen wurden?
   - ☐ ________________________Tage
   - ☐ ________________________Wochen
   - ☐ ________________________Monate
   - ☐ ________________________Jahre

6. Wie viel Zeit ist vergangen vom ersten Arzt-Besuch bis zur Diagnose?
   - ☐ ________________________Tage
   - ☐ ________________________Wochen
   - ☐ ________________________Monate
   - ☐ ________________________Jahre

7. Haben Sie zum Zeitpunkt der Diagnose geraucht?
   - ☐ Nein
   - ☐ Ja. Wie viel? ____

8. Haben Sie zum Zeitpunkt der Diagnose eines der folgenden Medikamente eingenommen?
   - ☐ Kontrazeptive (Empfängnisverhütung)
   - ☐ Nichtsteroidale Antirheumatika (Aspirin, Ibuprofen, Diclofenac, Mefenaminsäure, Coxibe)
   - ☐ Andere____

Physician questionnaire: Feasibility and acceptance of CalproQuest in primary care

1. **Der Fragebogen CalproQuest war gut einsetzbar in der Sprechstunde.**

○ Trifft überhaupt nicht zu	○ Trifft wenig zu	○ Trifft eher zu	○ Trifft in hohem Mass zu

2. **Der zeitliche Aufwand für das Ausfüllen von CalproQuest ist vertretbar.**

○ Trifft überhaupt nicht zu	○ Trifft wenig zu	○ Trifft eher zu	○ Trifft in hohem Mass zu

3. **Der Fragebogen CalproQuest war nützlich für die weitere Diagnosestellung.**

○ Trifft überhaupt nicht zu	○ Trifft wenig zu	○ Trifft eher zu	○ Trifft in hohem Mass zu

4. **Der Fragebogen CalproQuest hat mein diagnostisches und therapeutisches Prozedere positiv beeinflusst.**

○ Trifft überhaupt nicht zu	○ Trifft wenig zu	○ Trifft eher zu	○ Trifft in hohem Mass zu

5. **Ich werde den Fragebogen CalproQuest auch weiterhin in der Sprechstunde einsetzen.**

○ Trifft überhaupt nicht zu	○ Trifft wenig zu	○ Trifft eher zu	○ Trifft in hohem Mass zu

6. **Meine Patienten hatten keine Verständnisprobleme bei der Beantwortung des Fragebogens CalproQuest.**

○ Trifft überhaupt nicht zu	○ Trifft wenig zu	○ Trifft eher zu	○ Trifft in hohem Mass zu

7. **Bei Patienten mit anhaltenden Bauchbeschwerden veranlasse ich standardmässig eine Calprotectin-Bestimmung, brauche daher den Fragebogen CalproQuest nicht.**

○ Trifft überhaupt nicht zu	○ Trifft wenig zu	○ Trifft eher zu	○ Trifft in hohem Mass zu

Patient questionnaire: Acceptance of stool sampling

Sie leiden seit einiger Zeit an Bauchbeschwerden und haben deshalb ihren Hausarzt aufgesucht und eingewilligt, an der ALERT Studie teilzunehmen. Um die Gründe Ihrer Bauchbeschwerden herauszufinden hat ihr Hausarzt Sie gebeten, zu Hause Stuhlproben zu nehmen.
Bitte beantworten Sie uns ein paar Fragen hierzu.

1. Ich habe verstanden, wozu die Stuhlentnahme dient.

Trifft überhaupt nicht zu	Trifft wenig zu	Trifft eher zu	Trifft in hohem Mass zu

2. Ich hatte keine Mühe mit der Vorstellung, selber von meinem Stuhl eine Probe zu entnehmen.

Trifft überhaupt nicht zu	Trifft wenig zu	Trifft eher zu	Trifft in hohem Mass zu

3. Der Vorgang der Stuhlentnahme war schwierig für mich.
(Falls dies zutrifft, bitte kurz erläutern, wo die Schwierigkeiten lagen)

____

Trifft überhaupt nicht zu	Trifft wenig zu	Trifft eher zu	Trifft in hohem Mass zu

4. Ich würde wieder eine Stuhlprobe entnehmen, wenn es hilft, die richtige Diagnose zu stellen .

Trifft überhaupt nicht zu	Trifft wenig zu	Trifft eher zu	Trifft in hohem Mass zu

326-07-2014

Schlittler Gabriela

Von: Nadine Zahnd [nadine.zahnd@centerview.ch] im Auftrag von Nadine Zahnd [nadine.zahnd@ibdnet.ch]
Gesendet: Dienstag, 15. Juli 2014 13:39
An: Schlittler Gabriela
Cc: Rosenberger Tobias
Betreff: KEK-ZH-Nr. 2013-0516
Anlagen: 2013-0516.pdf

Kennzeichnung: Zur Nachverfolgung
Kennzeichnungsstatus: Gekennzeichnet

Kantonale Ethikkommission Zürich
Prof. Dr. med. Erich W. Russi
Präsident Abteilung B
Stampfenbachstrasse 121
8090 Zürich

Erhalten
Kenntnisnahme
25. Juli 2014

Sehr geehrte Frau Schlittler

Im Namen des Studienteams bedanke ich mich herzlich bei Ihnen für Ihren positiven Entschied.

Ich hatte Sie am 27.6.14 telefonisch kontaktiert, weil im Protokoll bei den Ein-/Ausschlusskriterien eine Spezifizierung vergessen gegangen ist: Die Studie besteht aus zwei Teilen A und B, wobei nicht jedes Ein-/Ausschlusskriterium auf beide Teile zutreffen. Wie telefonisch mit Ihnen und Herrn Rosenberger besprochen möchte ich Ihnen diese Spezifizierungen per Mail kommunizieren (Änderungen zum bewilligten Protokoll sind rot markiert):

Patients will be eligible if they
- Are ≥18 years old (Part A, B)
- Are referred to their gastroenterologist for any endoscopic examination (Part A)
- Visit their family doctor because of on-going unspecific gastrointestinal symptoms (abdominal pain, bloating, stool irregularities, chronic diarrhea) for at least two weeks (Part B)
- Underwent no further diagnostic procedures (endoscopy) for the current episode (Part B)

Patients will not be eligible, if they
- Are younger than 18 years (Part A, B)
- Have known abdominal pathologies (Part A, B)
- Had previous abdominal surgeries (Part B)
- Have been treated with steroids (topical and/or oral) and/or aminosalicylates within 30 days prior inclusion into this study (Part B)
- Underwent endoscopic examination within 3 years prior screening (Part B)

Ich bedanke mich ganz herzlich für Ihre Kenntnisnahme, wünsche Ihnen schöne Sommertage und grüsse Sie freundlich
Nadine Zahnd

Please note: I'm absent from July 18th trough August 4th with very limited e-mail access. In urgent cases please refer to my mobile number.

Dr. Nadine Zahnd-Straumann
Projektleitung ALERT

T +41 79 766 65 58
M nadine.zahnd@ibdnet.ch

IBDnet
c/o Stephan Vavricka
Stadtspital Triemli
Birmensdorferstrasse 497
