# Supplementary material for: Can the CalproQuest predict a positive Calprotectin test? A prospective diagnostic study
Source: PLoS One. 2019 Nov 21;14(11):e0224961. doi: 10.1371/journal.pone.0224961 (PMC6872045; doi:10.1371/journal.pone.0224961)
Supplement: S1 Fig — Consolidated Standards of Reporting Trials. (DOC) [file pone.0224961.s006.doc]

**
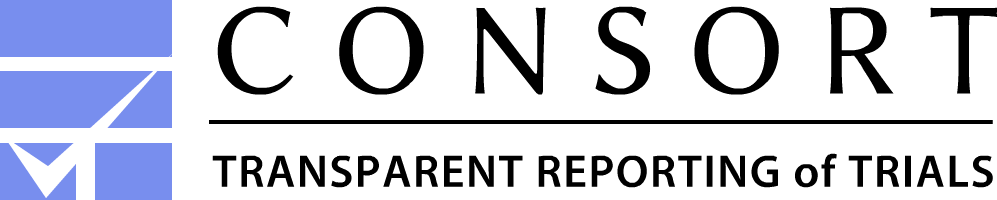
**

**CONSORT 2010 Flow Diagram**

**Allocation**

**Follow-Up**

**Enrollment**

Assessed for eligibility (n=191)

Excluded (n=3)

♦  Not meeting inclusion criteria (n=3)

♦  Declined to participate (n= 0)

♦  Other reasons (n= 0)

**See also Figure 1 of the Manuscript for Corresponding numbers.**

**Analysis**

Analysed (n=150)
♦ Excluded from analysis (give reasons) (n= 0)

Lost to follow-up (n= 25 missing Calprotectin tests, no reasons available)

Discontinued intervention (n= 6 missing Endoscopies, no reasons available)

Allocated to intervention CalproQuest (n= 188)

♦ Received allocated intervention (n=181 completed CalproQuests )

♦ Did not receive allocated intervention (give reasons) (n=7 incomplete CalproQuests)
